# Pramipexole restores depressed transmission in the ventral hippocampus following MPTP-lesion

**DOI:** 10.1038/srep44426

**Published:** 2017-03-14

**Authors:** Javier Castro-Hernández, Paul A. Adlard, David I. Finkelstein

**Affiliations:** 1 The Florey Institute of Neuroscience and Mental Health, Melbourne, Victoria 3052, Australia

## Abstract

The hippocampus has a significant association with memory, cognition and emotions. The dopaminergic projections from both the ventral tegmental area and substantia nigra are thought to be involved in hippocampal activity. To date, however, few studies have investigated dopaminergic innervation in the hippocampus or the functional consequences of reduced dopamine in disease models. Further complicating this, the hippocampus exhibits anatomical and functional differentiation along its dorso-ventral axis. In this work we investigated the role of dopamine on hippocampal long term potentiation using D-amphetamine, which stimulates dopamine release, and also examined how a dopaminergic lesion affects the synaptic transmission across the anatomic subdivisions of the hippocampus. Our findings indicate that a 1-methyl-4-phenyl-1, 2, 3, 6-tetrahydropyridine induced dopaminergic lesion has time-dependent effects and impacts mainly on the ventral region of the hippocampus, consistent with the density of dopaminergic innervation. Treatment with a preferential D_3_ receptor agonist pramipexole partly restored normal synaptic transmission and Long-Term Potentiation. These data suggest a new mechanism to explain some of the actions of pramipexole in Parkinson´s disease.

Dopamine (DA) exerts actions on motor activity, motivation, reward, emotion and cognition and is synthesized by dopaminergic neurons in multiple areas of the brain. Of particular interest to this study are the substantia nigra (SN) and ventral tegmental area (VTA), whose axons innervate different brain regions through the mesolimbic and nigrostriatal systems. The SN projects mainly to the dorsal caudate-putamen (CPu), and to a lesser extent to cortical structures, hippocampus, amygdala and subthalamic nucleus^1^,^2^. The VTA projects primarily to the prefrontal cortex, anterior cingulate cortex and anterior temporal structures such as the amygdala, the entorhinal cortex and hippocampus^3^. Dopamine is known to regulate many electrical and biochemical aspects of neuronal function including excitability, synaptic transmission, integration and plasticity^4^. There has been renewed interest in the role of DA in the hippocampal and cortical areas involved in cognition as it has become apparent that people within the Parkinson’s Disease spectrum invariably have cognitive impairment^5^.

The hippocampus is a complex formation located in the temporal lobe and is involved in memory. Whilst the hippocampus is generally considered as a homogeneous structure, increasing evidence points to the existence of differences in gene expression, function and anatomy along its dorso-ventral axis. Some authors suggest that dorsal hippocampus (dHip) is more related to different types of memory and cognitive functions, while ventral hippocampus (vHip) is more associated to stress, including depression and anxiety, emotion and reward^6^,^7^. Strange and co-workers propose a more complex model of organization where long-axis gradients are superimposed on discrete functional domains^8^.

One of the mechanisms for learning and memory consolidation in the hippocampus is long-term potentiation (LTP), which can induce de novo spine formation and enhance synaptic transmission^9^,^10^,^11^. Dopamine can also modulate synaptic plasticity within the hippocampus^12^,^13^, but the mechanisms involved and the role of DA in memory function/formation are poorly understood, in part because of the diffuse dopaminergic innervation and the low DA levels present in this structure. There is consensus though, that whilst the DA levels in the hippocampus are relatively low when compared to structures such as the CPu, they are higher in vHip than dHip in rodents^14^. This is important as synchronized DA transmission is crucial for several types of memory^15^ and for LTP induction in vHip^13^,^16^.

It is likely, therefore, that DA and its regulation may be important in variety of hippocampal functions. This is particularly relevant for conditions such as Parkinson’s Disease which is characterized by the loss/dysfunction of midbrain dopaminergic neurons with associated deficits in fine movement control and motor learning. In the last few years, however, there has been a growing interest in the study of non-motor symptoms such as emotional disturbances and cognitive decline, whose causes have remained largely unknown. Clinical and experimental findings have shown the existence of cognitive decline from early stages, and even before Parkinson’s Disease is diagnosed. Whereas research on non-motor symptoms has mainly focused on frontostriatal functions, several studies in the last 20 years found hippocampal atrophy in Parkinson’s Disease patients, some of which presented with memory impairment^17^. Depression and anxiety, mood disorders which have been related to vHip disturbances, are highly prevalent in Parkinson’s Disease patients^18^. A recent cohort study involving a half million people, concluded that there is a direct correlation between depression and the risk of developing Parkinson’s Disease^19^. Despite of the importance of these findings to the quality of life of people living with Parkinson’s Disease and the evidence of the importance of the vHip, little is known about the mechanisms underlying this.

The neurotoxin 1-methyl-4-phenyl-1, 2, 3, 6-tetrahydropyridine (MPTP) is a commonly used animal model for parkinsonism. This model causes a degeneration of the dopaminergic system, depleting DA and their metabolites in dorsal striatum and prefrontal cortex. However, due to the difficulty of measuring DA in hippocampus and the question of which region was studied, the effects are controversial. Some studies found no decrease in DA levels after MPTP administration^20^ while others showed a depletion after multiple MPTP injections^21^,^22^. Despite this uncertainty, MPTP intoxication has been shown to impair several types of memory and also damages synaptic transmission in mice^22^,^23^.

In this study we used electrophysiological techniques to investigate how DA pathways modulate hippocampal-dependent functions, using D-amphetamine (Amph) and R-pramipexole (PPX), a dopamine agonist in clinical use in Parkinson’s Disease. We also utilized the MPTP model of Parkinsonism in order to examine the effect of dopaminergic denervation on hippocampal function. We demonstrate that pharmacological elevation of DA with Amph results in an increase in LTP, depression of the Input/Output (I/O) curve and an augmentation in Paired Pulse Facilitation (PPF) ratio under both baseline and MPTP lesion conditions. These effects were consistent across the hippocampus, although larger in vHip than dHip. Moreover, PPX treatment succeeded in restoring the already depressed I/O curve of MPTP-lesioned mice and increased LTP and reduced the PPF ratio in vHip. These findings provide insight into the cognitive deficits and mood disorders that affect Parkinson’s Disease patients and partially explain the antidepressant properties previously described for PPX.

## Results

### LTP varies between the dorsal and ventral sections of the hippocampus

**Figure 1:**
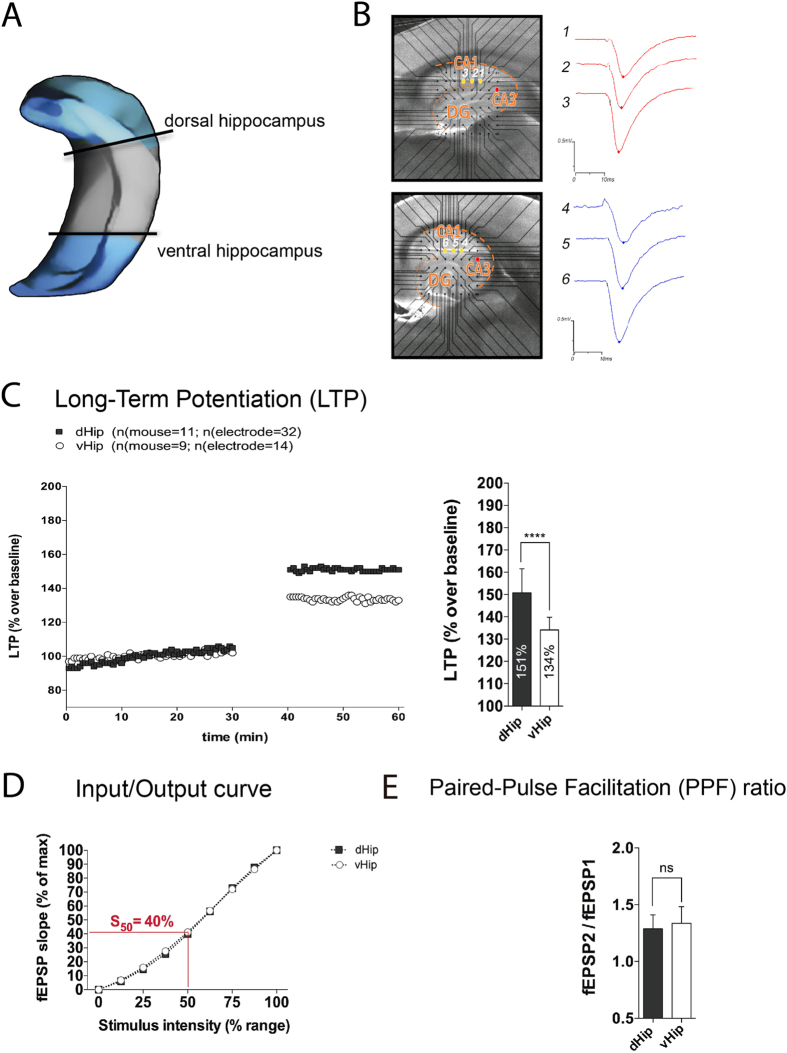
Electrophysiology experiments using the MEA system to study the differences between dorsal and ventral hippocampus. (**A**) Scheme of hippocampal dorso-ventral axis, and a slice sample of dorsal and ventral hippocampus placed into a MEA chip. Blue lighted zones were used in our study, avoiding the intermediate level of hippocampus. (**B**) Representative hippocampal slices from dorsal and ventral subregion. Red (stimulating electrode) and yellow (recording electrodes). Original traces from fEPSP are shown (**C**) HFS-induced LTP in vHip and dHip (Unpaired t-test, ****p < 0.0001) (**D**) I/O curve for studying the synaptic excitability differences between dHip and vHip (Unpaired t-test at 50% of maximum stimulation, S_50_ not significant difference). (**E**) PPF ratio indicating the probability of neurotransmitter release in dHip and vHip (Unpaired t-test, not significant difference). I/O curves and PPF ratio were recorded before high frequency stimulation.

To investigate the heterogeneous nature of the hippocampus, we selected sections from the dHip and vHip (Fig. 1A and B).

High frequency stimulation of the Schaffer collateral pathway resulted in larger LTP in dHip than vHip (control dHip, 151% vs. control vHip, 134%, p ≤ 0.0001) (Fig. 1C) when recorded from the proximal stratum radiatum of CA1. This was not accompanied by a change in excitability (I/O curve) (Fig. 1D) or probability of neurotransmitter release (PPF ratio) (Fig. 1E). We chose the 50% of maximum response (S_50_) to compare the I/O curves in all experiments, since this was the threshold for PPF, baseline and LTP recording.

### Amphetamine effects are more pronounced in ventral hippocampus

**Figure 2:**
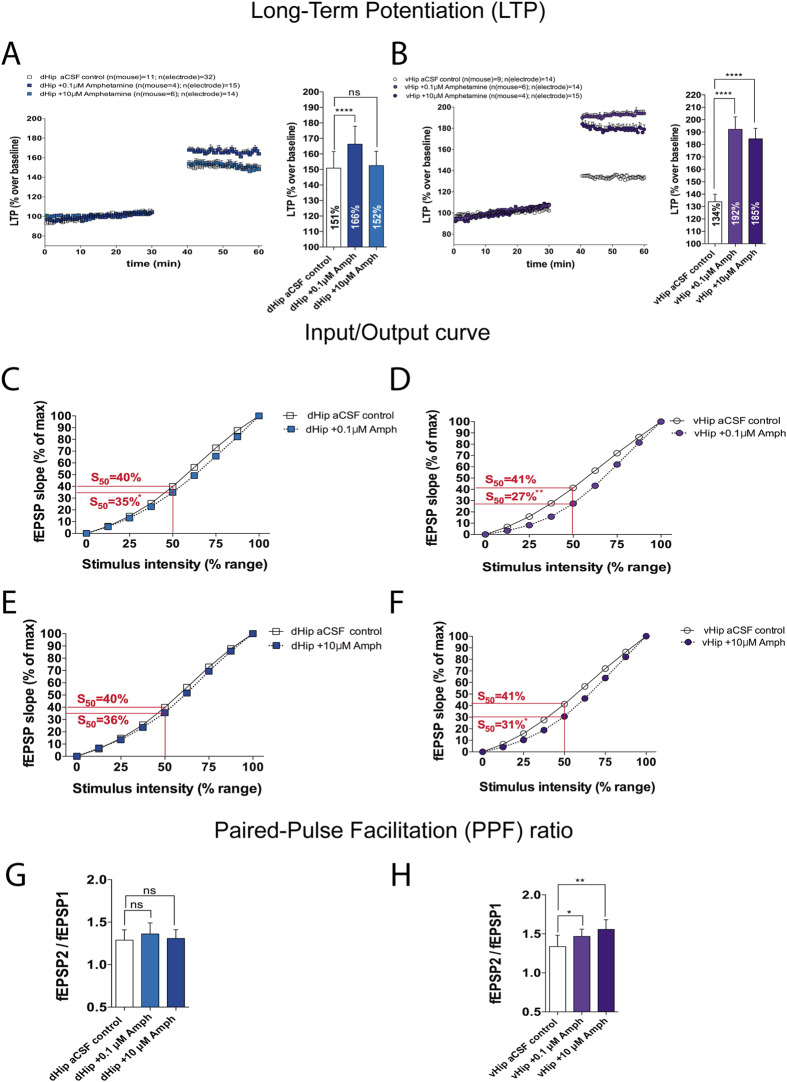
Effects of D-amphetamine along the dorso-ventral axis in hippocampus. (**A**) HFS-induced LTP in control dHip vs. 0.1 μM or 10 μM Amph (40 min before recording) (Dunnett´s post hoc test following a one-way ANOVA, *p < 0.0001) (**B**) HFS-induced LTP in vHip control vs. 0.1 μM or 10 μM Amph (40 min before recording). (Dunnett´s post hoc test following a one-way ANOVA, ****p < 0.0001). **(C)** I/O curve comparing dHip control vs. 0.1 μM Amph (Unpaired t-test at 50% of maximum stimulation, S_50_ **p < 0.05). (**D**) I/O curve comparing vHip control vs. 0.1 μM Amph (Unpaired t-test at 50% of maximum stimulation, S_50_ **p < 0.01). (**E**) I/O curve comparing dHip control vs. 10 μM Amph (Unpaired t-test at 50% of maximum stimulation, not significant difference). (**F**) I/O curve comparing vHip control vs. 10 μM Amph (Unpaired t-test at 50% of maximum stimulation, S_50_ *p < 0.05). **(G)** PPF ratio indicating the probability of neurotransmitter release in dHip control vs. 0.1 μM or 10 μM Amph (Dunnett´s post hoc test following a one-way ANOVA, not significant difference). (**H**) PPF ratio indicating the probability of neurotransmitter release in vHip control vs. 0.1 μM or 10 μM Amph (Dunnett´s post hoc test following a one-way ANOVA, *p < 0.05, **p < 0.01). I/O curves and PPF ratio were recorded before high frequency stimulation.

Amphetamine is a highly addictive psychostimulant that mainly acts through the dopamine transporter (DAT), the latter which controls DA reuptake from dopaminergic terminals and can modulate dopaminergic transmission^24^. Amphetamine reverses DAT transport and increases DA release^25^. We were interested in how this DA release modulates synaptic activity in hippocampus. A previous study found that DA caused an inverted-U shape action on cognition^26^, with both low dose and high doses resulting in degraded cognitive responses. Because of this we used two different concentrations (0.1 μM and 10 μM). Our results indicate that Amph evoked an increase in LTP in the both the dorsal and ventral hippocampus. When hippocampal sub-regions were analyzed we found that only the lower dose of Amph altered LTP in dHip (control, 151% vs. 0.1 μM Amph, 166%; p ≤ 0.0001; Fig. 2A). Both doses of Amph increased LTP in vHip (control, 134% vs. 0.1 μM Amph, 192%; p ≤ 0.0001; vs. 10 μM Amph, 185%; p ≤ 0.0001; Fig. 2B).

Synaptic excitability is directly determined by the frequency of activation, with excitable synapses producing more and larger action potentials at lower stimulation thresholds^4^. Aberrant excitatory activity of neurons has been described in Parkinson’s Disease. Counterintuitively, previous studies showed that Amph and other psychostimulants depressed the synaptic excitability of some brain structures like VTA and nucleus accumbens (NAc)^27^,^28^. In concordance with those studies, we found that Amph decreased the synaptic excitability in the hippocampus, but there were different effects along the dorsal and ventral slices. Amph had modest effects in the dHip, the lower dose produced a significant reduction (control, 40% vs. 0.1 μM Amph, 35%; p ≤ 0.05; Fig. 2C). However, vHip exhibited a more pronounced reduction after Amph exposure (control, 41% vs. 0.1 μM Amph, 27%; p ≤ 0.01; vs. 10 μM Amph, 31%; p ≤ 0.05; Fig. 2D and F).

As far as we know, the effect of Amph upon PPF in the different regions of the hippocampus has not been previously studied. However, a previous report showed that Amph increases the PPF ratio in the NAc^29^. Neuromodulators directly regulate the probability of neurotransmitter release from presynaptic boutons by a complex interplay of different presynaptic properties including the ability of altering the size and properties of the vesicle pool, the location of voltage-gated calcium channels at the active zone or the magnitude of calcium influx^30^. The data showed that Amph did not alter the PPF ratio in the dHip (Fig. 2G), but did decrease the probability of neurotransmitter release in the vHip (control PPF ratio, 1.37 vs. 0.1 μM Amph PPF ratio, 1.47; p ≤ 0.05; vs. 10 μM Amph PPF ratio, 1.56; p ≤ 0.01; Fig. 2H).

### Pramipexole enhances synaptic transmission in hippocampus

**Figure 3:**
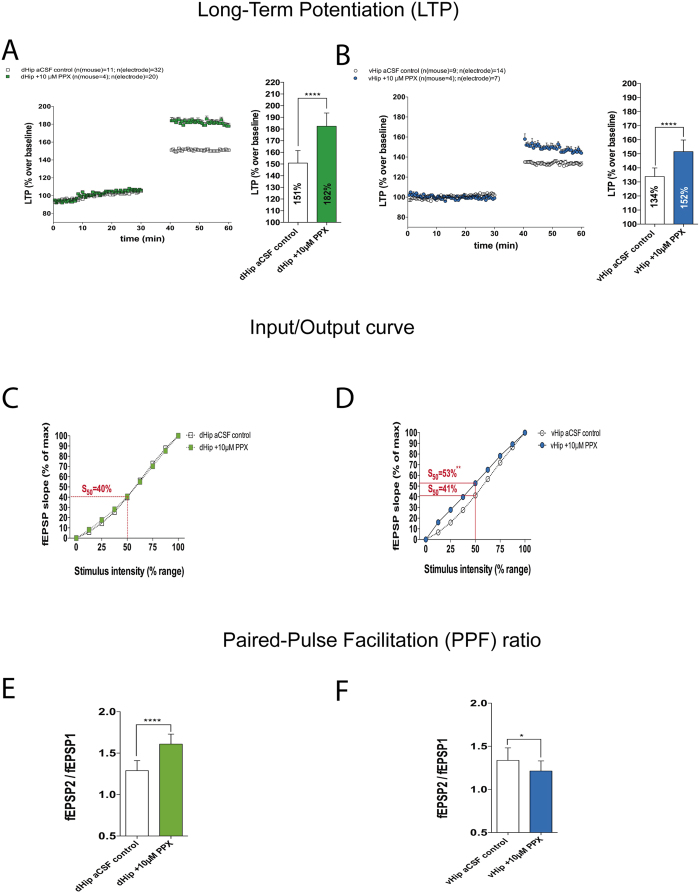
The effects of R-pramipexole treatment along the dorso-ventral axis in hippocampus. (**A**) HFS-induced LTP in dHip control vs. 10 μM PPX (40 min before recording) (Unpaired t-test, ****p < 0.0001). (**B**) HFS-induced LTP in vHip control vs. 10 μM PPX (40 min before recording) (Unpaired t-test, ****p < 0.0001). **(C)** I/O curve comparing dHip control vs. 10 μM PPX (Unpaired t-test at 50% of maximum stimulation, not significant difference). (**D**) I/O curve comparing vHip control vs. 10 μM PPX (Unpaired t-test at 50% of maximum stimulation, S_50_ **p < 0.01). (**E**) PPF ratio indicating the probability of neurotransmitter release in dHip control vs. after 10 μM (Unpaired t-test, ****p < 0.0001). (**F**) PPF ratio indicating the probability of neurotransmitter release in vHip control vs. after 10 μM PPX (Unpaired t-test, ****p < 0.0001). I/O curves and PPF ratio were recorded before high frequency stimulation.

PPX is a preferential D_3_ receptor agonist^31^. In contrast to the Amph-induced effect on LTP (Fig. 2), PPX enhanced LTP in the dHip (control, 151% vs. 10 μM PPX, 182%; p ≤ 0.0001, Fig. 3A), and vHip (control, 134% vs. 10 μM PPX, 152%; p ≤ 0.0001; Fig. 3B). These data agree with previous published data using a different D_3_-preferential agonist (7-OH-DPAT)^16^.

The effects of PPX on synaptic excitability were explored, and found not to alter it in the dHip (Fig. 3C). PPX had the opposite effect when compared with AMPH in the vHip (shown in Fig. 2D and F), evoking a significant increase in synaptic excitability (control, 41% vs. 10 μM PPX, 53%; p ≤ 0.01; Fig. 3D).

Moreover, PPX induced an increase in PPF in the dHip, indicating a decrease in probability of neurotransmitter release (control PPF ratio, 1.29 vs. 10 μM PPX PPF ratio, 1.61; p ≤ 0.0001; Fig. 3E). An increase in I/O curve and reduced PPF was found when PPX was administered to the vHip (control PPF ratio, 1.34 vs. 10 μM PPX PPF ratio, 1.21; p ≤ 0.05; Fig. 3F).

### The effects of recovery after MPTP lesion in ventral hippocampus

**Figure 4:**
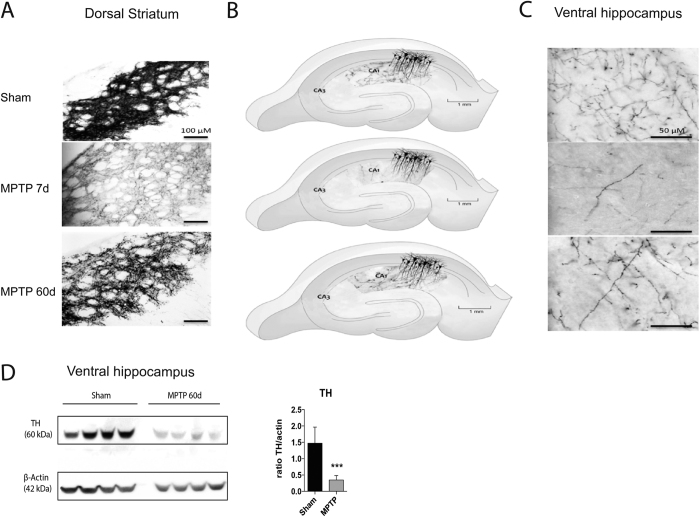
Effects of short- and long-term MPTP lesion on dopaminergic terminals in dorsal striatum and ventral hippocampus. (**A**) Immunohistochemistry for TH in dSt of sham mouse (upper), after 7 days of MPTP lesion (middle) (80% decrease), and after 60 days of MPTP lesion (down) (scale bar 100 μm). (**B**) Scheme of hippocampal sub-regions showing immunohistochemistry in CA1 of sham mouse (upper), after 7 days of MPTP lesion (middle), and after 60 days of lesion (down). **(C)** vHip of sham mouse (upper), after 7 days of MPTP lesion (middle), and after 60 days of MPTP lesion (down) (scale bar 50 μm). **(D)** Western-blot for TH and β-actin in vHip of sham mouse vs. vHip after 60 days of MPTP lesion (ratio TH/actin, Unpaired t-test, ***p < 0.001).

Stereological cell counts of the SNpc revealed that MPTP injections caused a 35% loss of nigral neurons (control, 5989 ± 72 SEM, n = 12; MPTP, 3897 ± 148, n = 21; p < 0.0001), and 28,7% loss of VTA (control, 5375 ± 206 SEM, n = 7; MPTP, 3732 ± 223 SEM, n = 5; p < 0.0001). Our aim was to investigate short- and long-term effects of MPTP lesion in the hippocampus. Seven days after MPTP, TH immunolabelling showed a dramatic denervation of the dorsal striatum (Fig. 4A) and the vHip (Fig. 4C). Sixty days after the MPTP lesion the remaining neurons activated sprouting mechanisms to partially reinnervate these regions, and a modest recovery in the density of innervation in the striatum and vHip was observed (Fig. 4A and C, respectively). These new terminals had larger varicosities, consistent with synaptic plasticity in the striatum^32^. Western blot revealed that the compensatory sprouting was not enough to fully restore the TH levels at 60 days post-MPTP (Fig. 4D).

### MPTP lesion triggers an abnormal hippocampal LTP

**Figure 5:**
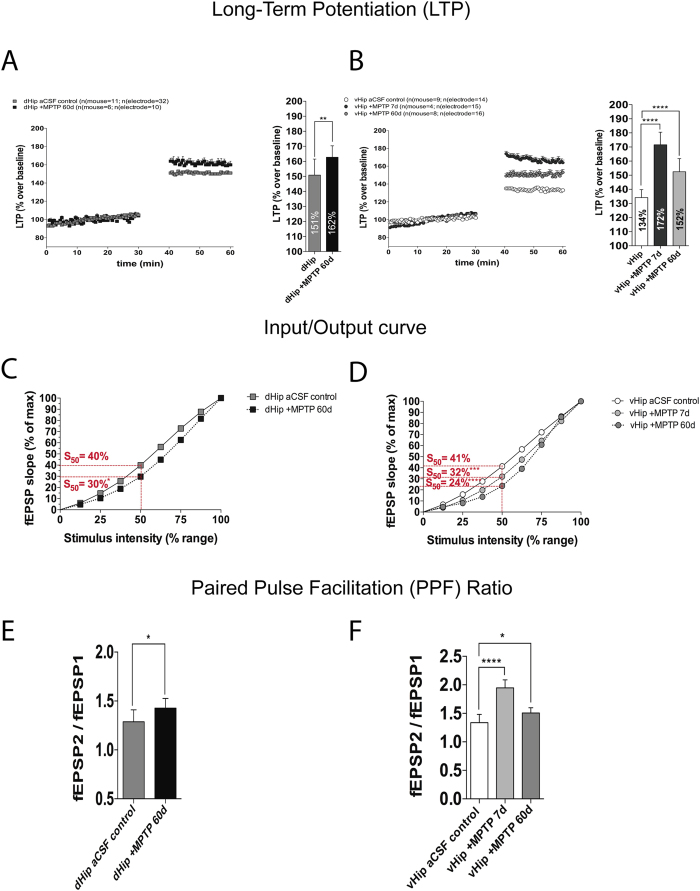
Electrophysiology experiments using MEA System to study the effects of short- and long-term MPTP lesion across the dorso-ventral axis in hippocampus. (**A**) HFS-induced LTP in dHip control vs. after 60 days of MPTP lesion (Unpaired t-test, **p < 0.01). (**B**) HFS-induced LTP in vHip control vs. after either 7 days or 60 days after MPTP lesion (Dunnett´s post hoc test following a one-way ANOVA, ****p < 0.0001). (**C**) I/O curve comparing dHip control vs. after 60 days of MPTP lesion (Unpaired t-test at 50% of maximum stimulation, S_50_ *p < 0.05). (**D**) I/O curve comparing vHip control vs. after either 7 days or 60 days of MPTP lesion (Dunnett´s post hoc test following a one-way ANOVA, ***p < 0.001, ****p < 0001). (**E**) PPF ratio indicating the probability of neurotransmitter release in dHip control vs. after 60 days of MPTP lesion (Unpaired t-test, *p < 0.05). (**F**) PPF ratio indicating the probability of neurotransmitter release in vHip control vs. after either 7 days or 60 days of MPTP lesion (Dunnett´s post hoc test following a one-way ANOVA, ****p < 0.0001, *p < 0.05). I/O curves and PPF ratio were recorded before high frequency stimulation.

We demonstrated that an MPTP nigral lesion increased LTP. When hippocampal sub-regions were separated, the data showed that dHip increased LTP (control, 151% vs. MPTP 60d, 162%; p ≤ 0.01; Fig. 5A). The vHip normally has a greater dopaminergic innervation, 7 days after the MPTP lesion we found enhanced LTP (control, 134% vs. MPTP 7d, 172%; p ≤ 0.0001; Fig. 5B). Sixty days after the MPTP lesion, LTP was still augmented (control, 134% vs. MPTP 60d, 152%; p ≤ 0.0001; Fig. 5B). These data suggest that the reduction of the dopaminergic innervation caused by MPTP lesion results in enhanced LTP, which is only partially reversed by reinnervation. These data suggest that dopamine innervation is required for normal LTP.

To investigate the function of these newly created synapses we explored synaptic excitability (I/O curve) and PPF. Interestingly, we found that MPTP lesion caused a drastic depression in excitability (I/O curve) throughout the whole hippocampus. After 60 days in dHip (control, 40% vs. MPTP 60d, 30%; p ≤ 0.05; Fig. 3C). This excitability gradually decreased over time in the vHip (control, 41% vs. MPTP 7d, 32%; p ≤ 0.001; vs. MPTP 60d, 24%, p ≤ 0.0001, Fig. 3D). Moreover, MPTP lesion increased the PPF in dHip (control PPF ratio, 1.29 vs. MPTP 60d PPF ratio, 1.43; p ≤ 0.05; Fig. 3E) and vHip (control PPF ratio, 1.34 vs. MPTP 7d PPF ratio, 1.95; p ≤ 0.0001; vs. MPTP 60d PPF ratio, 1.51; p ≤ 0.05; Fig. 3F). Taken together these data suggest that even though synaptic remodelling mechanisms are triggered after MPTP lesion, these synapses are not fully functional.

### Amphetamine had opposite effects on LTP in the dorsal and ventral hippocampus after MPTP lesion

The majority of currently available treatments are aimed at restoring DA levels; the most common of which is the DA precursor, L-Dopa. As it is recognized that excess DA can trigger emotional and cognitive impairments^12^, we examined the effect of elevated DA on LTP in the MPTP-lesioned mouse model.

**Figure 6:**
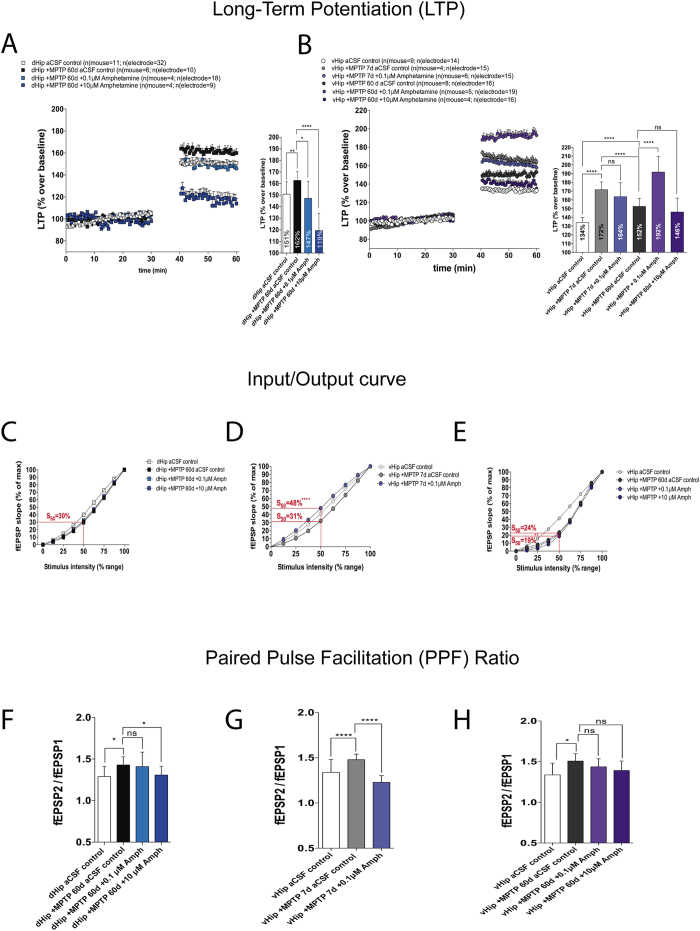
The effects of D-amphetamine on hippocampal synaptic plasticity of short- and long-term MPTP-lesioned mouse. (**A**) HFS-induced LTP in dHip vs. MPTP 60d (Unpaired t-test, **p < 0.01). dHip MPTP 60d vs. MPTP 60d 0.1 μM or 10 μM Amph (Dunnett´s post hoc test following a one-way ANOVA, * p < 0.05, **p < 0.01, ****p < 0.0001). (**B**) HFS-induced LTP in vHip vs. MPTP 7d (Unpaired t-test, ****p < 0.0001). vHip MPTP 7d vs. MPTP 7d 0.1 μM Amph (Unpaired t-test, not significant difference). vHip MPTP 60d vs. MPTP 60d 0.1 μM or 10 μM Amph (Dunnett´s post hoc test following a one-way ANOVA, ****p < 0.0001). (**C**) I/O curve dHip vs. MPTP 60d (Unpaired t-test at S_50_, not shown). dHip MPTP 60d vs. MPTP 60d 0.1 μM or 10 μM Amph (Dunnett´s post hoc test following a one-way ANOVA, not significant). (**D**) vHip vs. MPTP 7d (Unpaired t-test, S_50_ repeated data shown in Fig. 5). vHip MPTP 7d vs. MPTP 7d 0.1 μM Amph (Unpaired t-test at S_50_ not significant). vHip MPTP 7d vs MPTP 7d 0.1 μM Amph (Unpaired t-test at S_50_ ****p < 0.0001). vHip vs. MPTP 60d (Unpaired t-test at S_50_ shown in Fig. 5). vHip MPTP 60d vs. MPTP 60d 0.1 μM or 10 μM Amph (Dunnett´s post hoc test following a one-way ANOVA, not significant difference). (**E**) I/O curve comparing vHip vs. MPTP 60d (Unpaired t-test at S_50_, repeated data shown in Fig. 5). vHip MPTP 60d vs. MPTP 60d 0.1 μM or 10 μM Amph (Dunnett´s post hoc test following a one-way ANOVA, *p < 0.01). (**F**) PPF ratio in dHip vs. MPTP 60d (Unpaired t-test, *p < 0.05). dHip MPTP 60d vs. MPTP 60d 0.1 μM or 10 μM Amph (Dunnett´s post hoc test following a one-way ANOVA, *p < 0.05). (**G**) PPF ratio in vHip vs. MPTP 7d (Unpaired t-test, ****p < 0.0001). vHip MPTP 7d vs. MPTP 7d 0.1 μM Amph (Unpaired t-test, ****p < 0.0001). (**H**) PPF ratio in vHip vs. MPTP 60d (Unpaired t-test, *p < 0.05). vHip MPTP 60d vs. MPTP 60d 0.1 μM or 10 μM Amph (Dunnett´s post hoc test following a one-way ANOVA, not significant difference). I/O curves and PPF ratio were recorded before high frequency stimulation.

Amphetamine, which rapidly increases the levels of extracellular dopamine, caused opposite effects along the dorso-ventral hippocampal axis of MPTP-lesioned mice. Specifically, there was a reduction of LTP in the dHip (MPTP 60d control, 162% vs. MPTP 60d + 0.1 μM Amph, 147%; p ≤ 0.05; vs. MPTP 60d + 10 μM Amph 119%; p ≤ 0.0001; Fig. 5A) and an enhancement of LTP in the vHip (MPTP 60d 152% vs. MPTP 60d + 0.1 μM Amph, 192%; p ≤ 0.0001; Fig. 6B), the latter which is consistent with the dopaminergic terminal reinnervation shown in Fig. 4C. The main reason to perform the 7 day experiment after MPTP experiments was to check if the amphetamine–induced increase of LTP that we found in vH after 60 days of MPTP lesion was in response to the reinnervation and plasticity of the vH. Amphetamine did not induce any augmentation of LTP after 7 days in the vHip, (MPTP 7d control, 172% vs. MPTP 7d + 0.1 μM Amph, 164%; Fig. 6B). This data suggests that a reinnervation of hippocampus happened, partially recovering the ability for amphetamine to induce LTP.

Amphetamine could not further decrease the already depressed excitability of MPTP-lesioned mice in dHip (MPTP 60d control, 30% vs. MPTP 60d + 0.1 μM Amph, 32% vs. MPTP 60d + 10 Amph, 31%; not significant; Fig. 6C). However, Amph caused an increase in I/O curve of 155% after 7 days of MPTP lesion in the vHip (MPTP 7d control 31% vs. MPTP 7d + 0.1 μM Amph, 48%; p ≤ 0.0001; Fig. 6D). After 60 days, the higher dose of Amph further reduced (−21%) the suppressed I/O curve excitability in vHip (MPTP 60d control, 24% vs. MPTP 60d + 10 μM Amph, 19%; p ≤ 0.01; Fig. 6E), but the lower dose had no significant effect (MPTP 60d control, 24% vs. MPTP 60d + 10 μM Amph 22%; not significant; Fig. 6E).

Analysis of the PPF ratio indicated that only the higher doses of Amph increased the evoked potential in the dHip 60 days following MPTP lesion (MPTP 60d control PPF ratio, 1.43 vs. MPTP 60d + 0.1 μM Amph PPF ratio, 1.41 vs. MPTP 60d + 10 μM Amph PPF ratio, 1.31; p ≤ 0.05; Fig. 6F). In accordance with the change in I/O curve excitability, Amph produced a decrease in the PPF ratio in the vHip 7 days after MPTP lesion (MPTP 7d control PPF ratio, 1.48 vs. MPTP 7d + 0.1 μM Amph PPF ratio, 1.23, p ≤ 0.0001; Fig. 6G), but there were no changes in PPF ratio after 60 days indicating partial restoration of function (MPTP 60d control PPF ratio, 1.51 vs. MPTP 60d + 0.1 μM Amph PPF ratio, 1.44 vs. MPTP 60d + 10 μM Amph PPF ratio, 1.39; not significant; Fig. 6H).

### Pramipexole enhances the synaptic transmission in ventral hippocampus of MPTP-lesioned mice

**Figure 7:**
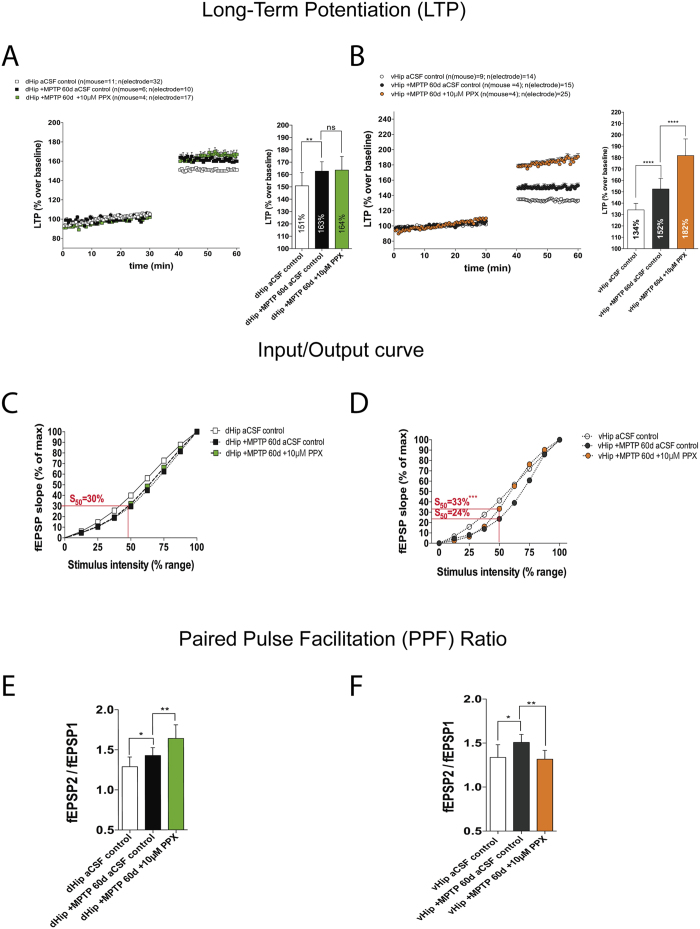
The effects of PPX on hippocampal synaptic plasticity of long-term MPTP-lesioned mouse. (**A**) HFS-induced LTP in dHip control vs. after 60 days of MPTP lesion (Unpaired t-test, **p < 0.01). dHip after 60 days of MPTP lesion vs. after 60 days of MPTP lesion treated with 10 μM PPX (Unpaired t-test, not significant difference). **(B)** HFS-induced LTP in vHip control vs. after 60 days of MPTP lesion (Unpaired t-test, ****p < 0.0001). vHip after 60 days of MPTP lesion vs. after 60 days of MPTP lesion treated with 10 μM PPX (Unpaired t-test, ****p < 0.0001). (**C**) I/O curve comparing dHip control vs. after 60 days of MPTP lesion (Unpaired t-test at 50% of maximum stimulation, S_50_ data shown in Fig. 3). I/O curve comparing dHip after 60 days of MPTP lesion vs. after 60 days of MPTP lesion treated with 10 μM PPX (Unpaired t-test, not significant difference). (**D**) I/O curve comparing vHip control vs. after 60 days of MPTP lesion (Unpaired t-test at 50% of maximum stimulation, S_50_ data shown in Fig. 3). I/O curve comparing vHip after 60 days of MPTP lesion vs. after 60 days of MPTP lesion treated with 10 μM PPX (Unpaired t-test, ***p < 0.001). (**E**) PPF ratio indicating the probability of neurotransmitter release in dHip control vs. after 60 days of MPTP lesion (Unpaired t-test, *p < 0.05). PPF ratio indicating the probability of neurotransmitter release in dHip control vs. dHip after 60 days of MPTP lesion treated with 10 μM PPX (Unpaired t-test, **p < 0.01). (**F**) PPF ratio indicating the probability of neurotransmitter release in vHip control vs. vHip after 60 days of MPTP lesion (Unpaired t-test, *p < 0.05). PPF ratio indicating the probability of neurotransmitter release in vHip control vs. vHip after 60 days of MPTP lesion treated with 10 μM PPX (Unpaired t-test, **p < 0.01).

Recently published data concluded that PPX improves depression-like symptoms in Parkinson’s Disease^33^ and in animal models^34^. However, the mechanisms involved have not been well characterized. Our data showed that PPX evoked an augmentation of LTP only in vHip (MPTP 60d control, 152% vs. MPTP 60d + 10 μM PPX; 182%; p ≤ 0.0001; Fig. 7B), with no effect in dHip (MPTP 60d control, 163% vs. MPTP 60d + 10 μM PPX 164%, not significant; Fig. 7A).

PPX did not affect excitability of PPF in dHip (MPTP 60d control, 30% vs. MPTP 60d + 10 μM PPX, 32%; Fig. 7C), and evoked potential of PPF as shown in Fig. 7E (MPTP 60d control PPF ratio, 1.43 vs. MPTP 60d + 10 μM PPX PPF ratio, 1.64; p ≤ 0.01). In contrast, PPX largely recovered the I/O curve excitability that had been depressed by MPTP lesion by up to 80% of normal value in vHip (MPTP 60d control, 24% vs. MPTP at 60d + 10 μM PPX, 33%, p ≤ 0.001; Fig. 7D) and decreased the PPF in the vHip hippocampal sub-region (MPTP 60d control PPF ratio, 1.51 vs. MPTP 60d + 10 μM PPX PPF ratio, 1.32; p ≤ 0.01; Fig. 7F). These data suggest that PPX has a postsynaptic site of action as well as a possible therapeutic benefit in the reformed synapses.

## Discussion

In this study we investigated the role of dopaminergic pathways in the function of the dorsal and ventral hippocampus. These results confirm that the dopaminergic system is a key regulator of normal hippocampal function and the MPTP data suggests that the dopaminergic lesion has different effects on synaptic transmission along the dorso-ventral axis in the hippocampus, having more profound effects on the vHip where there is a larger dopaminergic innervation^14^. Furthermore, the MPTP lesion resulted in time-dependent changes in dopamine: a short-term dopaminergic denervation and longer-term compensatory mechanisms that resulted in dysfunctional synapses^35^,^36^.

**Figure 8:**
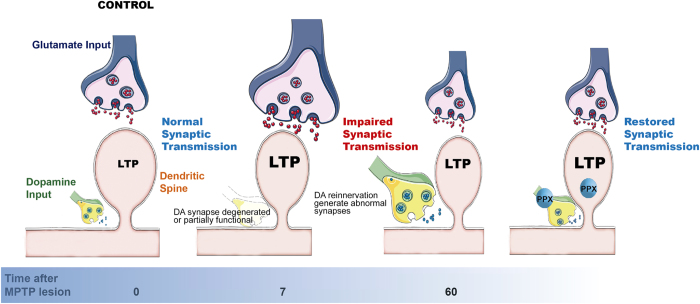
The triad: Dopamine, Glutamate and Spines Glutamatergic and DA terminals converge at a number of targets, including the frontal cortex, amygdala, NAc, dorsal and ventral striatum and hippocampus. Glutamatergic terminals are located, like caps, on the tops of the spines and the DA terminals synapse on the neck of the spines^62^. Regulation of this synaptic organization is now considered pivotal in understanding the synaptic plasticity that underpins CPu dysfunction in addiction and Parkinson´s disease^63^. However, these specialized structures have been poorly studied in hippocampus, where a recent study suggests that DA terminals also directly control GABA inhibitory interneurons. Our data and other studies suggest that, DA indirectly modulates pyramidal neurons activity^39^,^62^. We propose that seven days after MPTP dopaminergic terminal loss disrupted synaptic transmission, as the I/O curve decreased and PPF increased. After sixty days of lesion, LTP was downregulated by the newly innervated synapses. However, the synaptic activity was not fully restored. PPX succeeded on restoring the impaired synaptic transmission, and increasing LTP in MPTP-lesioned mouse.

Our results confirm that there is a differential capability to induce LTP across the dorso-ventral hippocampal axis. In agreement with these results we found that under normal conditions, LTP is larger in the dHip than vHip as have been described previously^37^. This phenomenon can be partly explained by differences in density of dopaminergic innervation along the hippocampal dorso-ventral axis^14^. The question of how this difference could be influenced in a pathological condition such as Parkinson’s Disease however, has yet to be answered. To address this question, we used a neurotoxin (MPTP) that preferentially causes a degeneration of dopaminergic neurons. We were interested in the short-term, but also particularly in the long-term effects of the lesion as this is more comparable to the type of chronic degeneration and compensatory repair that characterizes Parkinson’s Disease. We hypothesize that under normal conditions the dopaminergic innervation suppresses hippocampal excitability and LTP. During the course of Parkinson’s Disease the altered dopaminergic innervation results in hippocampal dysfunction and may partially explain some of the enhanced neuropsychiatric manifestations that occur in the disease. We found a distinct increase in LTP along the dorso-ventral hippocampal axis that corroborated our hypothesis. However, following MPTP lesion, the enhancement of LTP was more pronounced in vHip than dHip, coincident with the proportions of dopaminergic innervation. Interestingly, LTP induction after MPTP lesion was time-course dependent. Seven days after MPTP lesion, we found a robust increase in LTP in the vHip and the largest dopaminergic denervation. At 60 days post-MPTP lesion, LTP was still larger than control in both vHip and dHip, but downregulated compared with the short-term lesion in vHip. This moderation of LTP occurred in parallel with the dopaminergic reinnervation of vHip (Fig. 8). Also, we found that MPTP lesion caused a time-dependent depression in synaptic excitability, as indicated by alterations in PPF and the I/O curve. Recent works suggesting that a dopaminergic lesion could potentiate GABA inhibition could explain this phenomenon, but further experiments are needed^38^.

A theoretical model explains LTP as a mechanism for short term modification of the synapse and a longer term mechanism for stimulating spine formation^9^. Glutamate release is required to induce LTP, and induces de novo growth of functional spines^11^. Dopamine release from the VTA and SN inputs to the hippocampus are required for LTP at Schaffer collateral synapses where it controls the excitability of CA1 pyramidal neurons through direct modulation of GABAergic interneuron excitation^39^.

Moreover, it has been demonstrated that the late phase of hippocampal LTP (after 60 min of HFS) can be blocked in presence of D1 antagonists or D1 KO mice^40^,^41^,^42^,^43^,^44^. Although studies about synaptic transmission in hippocampus following MPTP lesion did not find significant differences of LTP from longer time of 30 min. post HFS or TBS^22^,^45^,^46^,^47^, further studies are needed to check whether our results are only related to the short phase of LTP.

We suggest a model (Fig. 8) where a short-term lesion causes an enhanced LTP as direct result of the loss of dopamine innervation^23^. There is a discordance amongst the studies that have examined LTP following MPTP lesion, which is most likely caused by different experimental approaches e.g. varying timeframes post-MPTP, different schedule of MPTP treatment, different protocol to induce LTP and different origins/orientations of the slices along the dorso-ventral axis of the hippocampus^22^,^47^,^48^. No previous studies have differentiated between dHip and vHip^22^,^45^,^46^,^47^. Further other studies found an enhancement of fEPSP when MPTP was applied to the hippocampus in the bath^23^. The theoretical model explains LTP as a mechanism for short term modification of the synapse and in the longer term a mechanism for stimulating spine formation. Other authors have found enhanced LTP induced by an injury. Injury of visual cortex is followed by processes of enhanced neuroplasticity like LTP^49^. Other studies found an increase in LTP was observed after closed head injury in hippocampal CA1^50^. These data support the view that that a compensatory mechanism for LTP or a mechanism of remodeling the neuronal networks is plausible following injury. Our results indicate that function is gradually and only partially restored in 60 days when the hippocampal DA networks are gradually recovered. This suggests that these newly formed terminals only partially conserve the normal function of DA networks, and this could be aided by using drugs that modulate dopaminergic actions (Amph-like or PPX).

Amphetamine is a commonly used cognitive enhancer prescribed for attention-deficit hyperactivity disorder (ADHD). It is known that Amph promotes LTP in CA1 region of the hippocampus^51^. Whilst the cellular response to Amph depends on the level of DAT expression, with its activity and potency varying across striatal sub-regions^52^, the mechanisms underlying the differential effects of Amph in the hippocampus has yet to be clarified. Therefore, we sought to determine whether Amph had a larger effect in vHip because of the larger dopaminergic innervation or a because of differential effects on cathecolamines and serotonin^24^. Our data indicate that Amph enhances LTP in the whole hippocampus, but its potency varies along the dorso-ventral axis, with the vHip showing the largest increase in LTP. Also, we found that Amph depressed the synaptic excitability in the vHip, as previously described in NAc^28^ and VTA^27^. Moreover, Amph increased the PPF in the vHip, similar to that shown in the NAc^29^. Amph could also have dopamine independent effects, that is, acting directly on serotonin receptors or cannabinoid receptor type 1^27^,^53^.

We used multiple methods to assess the effect of the lesion; stereology of SNpc neurons (assessed as a 35% lesion); stereology of VTA neurons (29% lesion), TH immunohistochemistry of CPu (80% decrease in densitometry); western blot of hippocampal slices for tyrosine hydroxylase (show 75% decrease in TH that persists even up to 60 days, Fig. 4d). The appears to be no standard way to produce or assess dopaminergic lesions following a toxin lesion in rodents. The complexity includes; the dose, the method used to assess denervation and the timing of the assessment after intoxication^36^. Given these limitations, it appears that the lesions produced for this study are equivalent to those previous studies.

Utilising the MPTP-lesioned mice we confirmed that low dose Amph caused enhancement of LTP and this was dependent on the dopaminergic system. Seven days after MPTP lesion is coincident with profound dopaminergic denervation, Amph did not induce any LTP increase in the vHip. In contrast, after 60 days of MPTP lesion, when the dopaminergic innervation is partly restored, Amph did recover its ability to induce LTP. This data indicate that the dopaminergic system is crucial for low dose Amph-mediated LTP induction in the vHip. The observation that Amph caused a decrease in LTP in the dHip of MPTP-lesioned mice may explain cognitive and emotional alterations found in individuals treated with L-Dopa^12^. It is reasoned that Amph is acting as a DA releaser, increasing DA in the synaptic cleft to give the same functional result as current dopamine therapeutics. We want to emphasise an apparent parallelism between MPTP and Amph-induced LTP, where in both treatments synaptic excitability was depressed and PPF was increased in vHip.

We also examined the effects of PPX, a D_3_-preferent agonist, which was found to increase LTP in the hippocampus in a similar manner to that shown with another D_3_ dopamine receptor agonist (7-OH-DPAT)^16^. The differences with Amph are possibly due to D_3_R being expressed not only in the presynaptic dopaminergic neurons, but also in the postsynaptic neuron where it modulates GABAergic transmission^54^. However, further experiments using KO mice or antagonists are needed to check whether this effect is D_3_R-mediated. We also found that PPX evoked a disinhibition in vHip, as demonstrated by either a decrease in PPF ratio or by an increase in the synaptic excitability (I/O curve). Hammad and Wagner described this phenomenon using another D_3_ receptor agonist (PD 128907)^54^, although they did not separate slices along the dorso-ventral axis and our data indicate that this effect is vHip specific. This disinhibitory ability of PPX led us to question whether PPX could restore normal hippocampal function in MPTP-lesioned mice. Our results indicate that PPX succeeded, not only increasing the I/O curve and decreasing the PPF of MPTP-lesioned mice, but increasing the LTP in vHip. These data add a new mechanism of action for PPX, and could partly explain the antidepressant effects described for PPX in both Parkinson’s Disease^33^ and models of Parkinsonism^55^. Moreover, we propose a possible mechanism that could be considered to counteract the depressing effect caused by Amph and other psychostimulants^27^,^28^. However, whether this disinhibitory effect of PPX in the vHip could be used for restoring the depressed excitability induced by Amph and its impact over drug addiction should be further explored. Also, regarding to the mechanism of action of PPX, experimental studies carried out over the last two decades indicate that D_3_R agonists can mediate their neuroprotective effects through D_3_R-dependent and D_3_R-indepenendent mechanisms^56^,^57^. Therefore, further experiments would be needed to fully characterize all the dopaminergic receptors action in these models.

Our findings shed light on the role of dopaminergic system on hippocampal function, and particularly could help for the better understanding of the sequential mood changes associated with Parkinson’s Disease. The main findings are: 1) the dopaminergic system has a prominent effect on the synaptic activity in vHip but lesser in the dHip; 2) The discovery of a differential susceptibility to dopaminergic MPTP lesion across the dorso-ventral axis in hippocampus. These lesions have distinct acute and chronic effects; 3) We propose a novel mechanism of PPX that should be further explored for cognitive and mood disorders associated with Parkinson’s Disease.

## Methods

### Animals

Male C57BL/6 J mice aged 11 weeks weighing between 20 and 25 g were used for this study. All procedures involving mice conformed to the Australian National Health and Medical Research Council code of practice for the care and use of animals for scientific purposes and were approved by the Florey Institute animal ethics committee. All experiments were designed to minimize the number of animals used, pain and discomfort.

### MPTP Intoxication protocols

C57BL/6 J mice were administered MPTP (Sigma, USA) in an acute dosing regimen of 60 mg/Kg given over four injections (15 mg/Kg each) 2 hours apart^58^. Each experimental trial contained MPTP-lesioned animals that were randomly subdivided into a sham group and MPTP lesion group. Experimenters were blinded to the assignment for each of the groups. After either 7 or 60 days post-MPTP administration, mice were either culled and brains collected for LTP or they were deeply anaesthetized and tissues collected for either western blot or stereology^58^. The size of the lesion was assessed using stereology. The total number of DA neurons in the SNpc was estimated using a fractionator sampling design^32^,^58^,^59^. Counts were made at regular predetermined intervals (x = 140 μm, y = 140 μm). Systematic samples of the area occupied by the nuclei were made from a random starting point. An unbiased counting frame of known area (45 μm × 35 μm) was superimposed on the image of the tissue sections using stereology software (MBF, Stereo Investigator) utilizing a 63x objective lens (Leica, N.A. 1.36). Experimenters were blinded to the treatments of each of the groups. The dopamine axonal innervation of the dorsal striatum was assessed by measuring the densitometry of TH immunoreactivity using Image J (v 1.49, NIH).

### Microelectrode array electrophysiology

We study synaptic activity parameters such as; The I/O curve and PPF. The majority of hippocampal synapses terminate on dendrites that integrate with multiple inputs that compute to produce an output (field excitatory postsynaptic potential, fEPSP). I/O curve serves as an index of synaptic excitability of large neuronal populations^60^. PPF ratio (fEPSP2/fEPSP1) is used as an easy measure of the probability of neurotransmitter release, where an increase of PPF ratio is interpreted as a decrease of probability of neurotransmitter release, and vice versa^61^.

The animals were killed and brain removed. The brain was cut into 300 μm Horizontal sections with a vibratome (Leica VT1200S) according to the lamellar orientation of hippocampus. We chose slices belonging to dHip, that serves cognitive function, and the vHip that corresponds to the affective hippocampus. Intermediate slices were discarded as it has partly overlapping characteristics with its neighbours^7^ (see Fig. 1A). After sectioning, ventral or dorsal hippocampal slices were pre-incubated for 40 min at 34˚C with either D-amphetamine (0.1 μM or 10 μM; Sigma, USA, pramipexole (10 μM; Sigma) in carbogen bubbled aCSF solution, or carbogenated aCSF solution alone (control).

One acute hippocampal slice was transferred from slice-holding container (60 min pre-incubation in carbogenated aCSF at 34 °C) to a 3D-MEA chip with 60 electrodes spaced 200 μm apart (60 MEA 200/30 iR-Ti: MCS GnbH, Reutlingen, Germany). The slice was immobilized with a harp grid (ALA Scientific Instruments, New York, USA) and was continuously perfused with carbogenated aCSF (3 ml/min at 32˚C). The Schaffer-collateral pathway was stimulated by injecting a biphasic current waveform (100 μs) through one selected electrode at 0.033 Hz. Care was taken to choose the stimulating electrode in the same region from one slice to the other. The peak-to-peak amplitude of fEPSP at the proximal stratum radiatum of CA1 was analyzed using LTP-Analyzer (MCS GnbH, Reutlingen, Germany). Following a 20 min incubation period, slices were continuously stimulated with medium-strength stimuli. When stable evoked fEPSPs were detected (after at least 20 min), the stimulus threshold was determined, and a stimulus strength-evoked response curve (i.e. input-output, stimulation voltage vs evoked response amplitude) was recorded by gradually increasing stimulus intensity until the maximal fEPSP (peak amplitude of fESPS was appeared) was obtained. Only electrodes showing PPF were chosen for LTP stimulation and recording. The intensity of the test stimulus was set to be 50% of the threshold (S_50_). After recording at least 30 min of stable baseline fEPSPs (baseline control sequence), LTP was induced by applying 3 bursts of high frequency stimulus; 3 × 100 Hz, 500ms width with 20 s interval at the tested stimulation intensity, then fEPSPs were recorded for 30 min. For illustration purposes a 10 min interval from the tetanus, has not been displayed or used in the calculation of LTP. We have only shown the LTP response once stabilized. 20 to 80% of peak amplitude of the initial portion of fEPSP was analyzed (LTP data are expressed as % of fEPSPs over the baseline). After LTP recording, PPF (PPF2) was registered again in order to check the health of the slices (data not shown). PPF ratio was calculated by dividing the second peak of fEPSP amplitude by the first peak of fEPSP amplitude.

### Immunohistochemistry for Tyrosine Hydroxylase

In a different group of mice 30 μm horizontal sections were cut with a cryostat (Leica, Germany), collected in parallel series, and immersed for 30 minutes in 3% H_2_O_2_ to inactivate endogenous peroxidase, and incubated for 60 minutes at room temperature in 4% normal goat serum (NGS; Jackson ImmunoResearch, West Grove, PA) in PBS, containing 0.05% Triton X-100 (TX-100; Sigma), and overnight in PBS containing 2% NGS and rabbit anti-TH polyclonal antibody (1: 3000; Millipore, USA). After several rinses, the sections were incubated for 2 hours in biotinylated rabbit anti-goat antiserum (1:300; Sigma), and 1:200 NGS in PBS. Immunoreactions were visible after incubation for 1 hour at RT in ExtrAvidin–peroxidase (1:1500; Sigma) in PBS, and after 5 minutes in 0.005% 3′-3′-diamiobenzidine tetrahydrochloride (DAB; Sigma) and 0.001% H_2_O_2_ in cacodylate buffer 0.05 N pH 7.6. After several rinses, sections were mounted on gelatinized slides, dehydrated, coverslipped with DePeX and photographed with a Leica microscope (Leica, N.A. 1.36).

### Western-blot for Tyrosine Hydroxylase

Western blot was used to investigate protein expression of TH (1:10,000; Millipore), Samples were electrophoresed in a Bolt system using 4–12% Bis-Tris Plus 10 well gels (Thermo-Fisher, USA).

### Statistical analysis

A one-way ANOVA with Dunnett’s post-hoc test or unpaired t-tests were performed (Graphpad Prism, version 6, USA), with p < 0.05 considered statistically significant. All averaged results are expressed as mean ± SEM values.

## Additional Information

**How to cite this article:** Castro-Hernández, J. *et al*. Pramipexole restores depressed transmission in the ventral hippocampus following MPTP-lesion. *Sci. Rep.* **7**, 44426; doi: 10.1038/srep44426 (2017).

**Publisher's note:** Springer Nature remains neutral with regard to jurisdictional claims in published maps and institutional affiliations.
